# Evolution of Biologics Screening Technologies

**DOI:** 10.3390/ph6050681

**Published:** 2013-05-14

**Authors:** Peter Cariuk, Matthew J. Gardener, Tristan J. Vaughan

**Affiliations:** MedImmune Ltd, Milstein Building, Granta Park, Cambridge CB21 6GH, UK; E-Mails: GardenerM@MedImmune.com (M.J.G.); VaughanT@MedImmune.com (T.J.V.)

**Keywords:** high throughput screening, assay technologies, therapeutic antibodies

## Abstract

Screening for biologics, in particular antibody drugs, has evolved significantly over the last 20 years. Initially, the screening processes and technologies from many years experience with small molecules were adopted and modified to suit the needs of biologics discovery. Since then, antibody drug discovery has matured significantly and is today investing earlier in new technologies that commercial suppliers are now developing specifically to meet the growing needs of large molecule screening. Here, we review the evolution of screening and automation technologies employed in antibody discovery and highlight the benefits that these changes have brought.

## 1. Introduction

By the mid 1990s the advent of combinatorial chemistry required pharmaceutical companies to adopt assay miniaturisation, the use of robotics and simple “mix and measure” technologies to facilitate cost effective ultra high throughput screening of every individual small molecule contained within libraries in excess of 1 × 10^6^ compounds.

**Figure 1:**
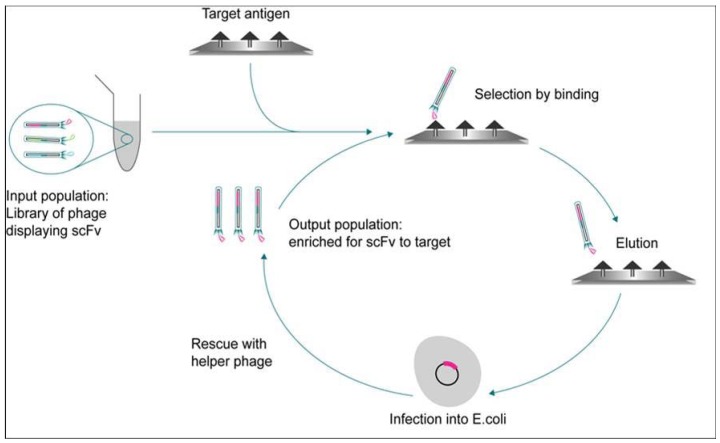
Schematic of a simple antibody phage display protocol.

In contrast, combinatorial human antibody libraries (scFv or Fab) made via phage display in *E. coli*, are significantly larger, containing as many as 1 × 10^11^ individual compounds [1]. As such, they are too large and diverse to be screened as individual members. Instead, the tremendous power of phage display selection [2] is used to identify a sub-population of antibodies from the 10^11^ library which are enriched for binding to the target of interest (Figure 1). These sub-populations are typically 10^4^–10^6^ and within the same size range as a small molecule library making them much more amenable to high throughput screening.

Two or three iterative cycles lead to an enrichment of binding variants with the desired specificity for the target antigen resulting in selected populations numbering 10,000–100,000. Typically, around 20%–70% of the antibodies in these selected populations recognize and bind their target antigen.

Historically, the outputs from antibody selections were assessed by simple ELISA [3] based methods in order to identify the strongest binders. These binding “hits” identified by ELISA were then profiled for the desired pharmacological characteristics, such as antagonism or agonism, firstly as purified antibody fragments and finally as intact IgG’s using cell based functional assays. The techniques available for these ELISAs were manual and in 96-well format only. This severely limited the throughput of the discovery process and would often result in failure to identify those antibodies that may be less common in the selected population but have the desired properties. In addition, detection methods such as HRP and alkaline phosphatase were employed which have limitations in terms of their dynamic range and where the upper limits of detection are dictated by instrument sensitivity.

The disadvantages of this process were obvious. Only a small number of the possible antibodies from the selection outputs could be sampled and a high binding signal by ELISA did not always indicate a potent functional antibody with the desired properties. In addition, standard plate based ELISA’s were limited to those antigens which could be purified and immobilised to solid surfaces and retain epitopes that are representative of their native conformation.

To overcome these inherent problems, and maximise the chances of isolating diverse panels of lead antibodies, it was necessary to adopt screening strategies which facilitated the testing of higher numbers of antibodies from selection outputs. Critically, the assays implemented should be more predictive of activity in downstream disease relevant, functional cellular assays.

## 2. Heterogeneous Biochemical Assay Formats

Initially there were two main aims for improving screening methodologies for biologics, Firstly to find antibodies with the desired function (not just binding) and secondly to reduce the time taken to identify such leads by deploying this as early as possible in the discovery assay cascade. Unlike small molecule libraries which are composed of purified individual samples, antibody libraries due to their greater size and complexity, can currently only be screened as non-purified samples in a background of *E. coli* proteins. Hence challenges which had to be overcome included the need for sample tolerance and assay sensitivity, due the media used for growing bacteria and low concentrations of expressed antibody fragments. Initial progress in screening came from the use of plate washers, dispensers and automated liquid handlers. This greatly increased the throughput enabling multiple (10–20) 96-well plates to be screened for each given selection output. In parallel, the replacement of simple binding ELISAs with ligand competition assays enabled the identification of functional antibodies to be preferentially identified. However, these assays still relied on HRP and alkaline phosphatase detection readouts, limiting the assay sensitivity.

The advent of Dissociation-Enhanced Lanthanide Fluorescent Immunoassays-(DELFIA^®^) [4] with readily available labelling kits for target antigens and detection reagents, meant it was quickly adopted as a suitable alternative to HRP and alkaline phosphatase. DELFIA^®^ allowed the development of very sensitive assays with a wider dynamic range than traditional ELISA-based approaches and became the first non radioactive, high throughput screening technology to be widely adopted. DELFIA^®^ assays were used in antibody discovery for both ligand and receptor based targets, as well as in lead optimisation for isolating antibody variants with higher affinities.

The introduction of heterogeneous radio-ligand assay formats such as Filter Plate assays [5] brought further benefits. In particular, it enabled the development of ligand-receptor binding and proliferation assays in 96-well format. Radio-labelled ligands, both from commercial sources and via custom labelling, facilitated antibody screening against additional target classes such as G-protein coupled receptors (GPCR’s). However, the throughput remained restrictive due to limitations with radioactive material storage, and the need to have automation dedicated to radioactive work. Custom labelling of specific reagents could also add a significant cost to the overall assay development and screening.

Although a significant improvement over binding ELISAs, these heterogeneous assay formats were not yet ideal due to various factors such as prolonged incubation times, numerous wash steps and potential quenching of signal from bacterial extracts. It was important to ensure the wash steps were very thorough in order to remove unbound Europium and or radio-ligand and avoid the generation of “hot-spots” on the assay plates.

## 3. Homogeneous Biochemical Assay Formats

Homogeneous radiometric assay formats such as the FlashPlate^®^ [6] offered several advantages over the filter plate assay methods such as miniaturisation in 384 well format. However, for the best results, the plates still required blocking and wash steps. The subsequent introduction of Scintillation Proximity Assay (SPA) [7] technology enabled radiometric assays to be performed in a homogeneous mix and measure format where binding measurements without separation could be achieved. SPA provided the user with flexibility in assay design, a reduction in the quantity of radioactive labelling required and the ability to optimize the sensitivity of the assay by altering the quantity of SPA beads. The homogenous assay format offered distinct advantages over heterogeneous assay formats in terms of throughput and assay simplicity, although the use of radio-labels still presented significant health and safety, logistical and cost implications.

A major step forward in the high throughput screening of biological entities came with the advent of homogeneous time resolved FRET assays such as LANCE^®^ and HTRF^®^ [8,9] coupled with a tool box of reagents and labelling chemistries, Many of the heterogeneous assay formats used for studying ligand-receptor interactions were easily adapted to simple mix and measure homogeneous assay formats which could also be miniaturised to 384 well format, allowing an increase in throughput. These assays were also tolerant of crude bacterial supernatants from *E. coli*, crucial for the screening of antibodies from phage display libraries.

The homogeneous time resolved FRET assays were quickly adopted as screens to readily identify antibody variants with higher affinity relative to a lead, parental antibody. The lead antibody was easily labelled with a lanthanide for use in such competition screens. In addition, the AlphaScreen™ technology [10], was adopted for screening of protein:protein interactions, especially those of relatively low affinity (<100 nM), due to the increased avidity seen with the high antibody or antigen coating density on beads leading to increases in assay sensitivity.

## 4. Cell Based Binding Assay Platforms

The screening methods described to date require purified target protein. This often hampers the study of some more complex targets, such as cell-surface receptors. These proteins can be difficult to purify and in addition may require auxiliary sub-units for functionality. There is increasing interest in the biologics arena to generate therapies to these relatively complex target antigens, leading to the development of high throughput cell-based binding assay platforms. HTRF technology has evolved to meet some of these challenges through the development of Tag-lite^®^ technology [11], allowing cell-based, homogeneous and non-radioactive ligand binding to be performed across a wide variety of GPCR receptor types coupled with functional assay readouts to measure second messengers and phosphorylated proteins [12].

The introduction of a fluorescence macro-confocal biological binding event analyzer (Applied Biosystems 8200 Cellular Detection System), enabled high throughput screening across a broad range of cell-based assays. Adherent and suspension cells may be tested in either a live or fixed state allowing the monitoring of surface as well as intracellular antigens. The homogeneous, multiplex and multiplate format (384 and 96 well compatible) of fluorometric microvolume assay technology (FMAT^®^) assays [13], made the technology suitable for high-throughput screening using both endogenous and recombinant adherent and non adherent cell lines. The main benefit was to enable the use of homogeneous ligand-binding assays on whole cells, replacing the need to perform radioligand binding and thus resulting in reduced hands-on time and reagent usage compared to radiometric techniques.

This technology became rapidly adopted for developing high throughput screens and binding assays. Although this particular automation solution is now discontinued, it can be replaced by alternative technologies such as the Mirrorball (TTP LabTech) which we have shown to offer similar sensitivity, ease of use and comparable data to the ABI 8200 FMAT^®^ instrument. The Mirrorball offers expanded capabilities such as simultaneous scanning with multiple lasers allowing the use of 405 nm, 488 nm and 640 nm excitable reagents thus enabling high levels of multiplexing where stains can be applied to distinguish cell types or report biological activity. In addition the IntelliCyt^®^ HTFC screening system offers a similar degree of functionality making it possible to conduct highthroughput flow cytometry using non-adherent cells or beads in either 96 or 384 well format. The technology is highly sensitive with a high dynamic range and allows the use of microliter volumes of cells or beads [14].

## 5. Functional Screening Assays

Maintaining proteins such as GPCRs and ion channels in an appropriate native conformational state (e.g., agonistic, inverse agonist, antagonist) upon purification is rarely attainable. This makes the high throughput screening on an intact cell surface expressing such receptors a vital component the assay cascade for these target classes. The ability to screen for antibody fragments that both bind and alter the function of cell-surface receptors, either by competing with a natural ligand or by acting allosterically is key to success. By far the greatest challenge is the lack of tolerance of many of the cell lines to the crude bacterial samples used when screening the selection outputs. For this reason, those assay formats with shorter readout times are generally most tolerant of crude bacterial samples. For example, coupling assay readouts to the recruitment of β-arrestin [15,16]. These assays are also relatively sensitive to antagonism. The homogeneous time resolved FRET cAMP assay systems represent a relatively sensitive screening platform for biologics. Due to differences in read-out times the possibility of multiplexing β-arrestin recruitment assays with either homogeneous time resolved FRET or FLIPR calcium release assays remains to be explored.

Looking forward, the use of high throughput purification technologies such as the Bravo liquid handler (Agilent) or Multitrap™ (GE Healthcare) could provide a way to purify samples and allow functional screens to be routinely used, but these are currently costly. Alternative expression hosts are currently being explored, such as mammalian expression systems and low endotoxin *E. coli* which may provide an advantage over bacterial expression systems due to lower levels of the interfering compounds.

## 6. Label Free Assays

Cell-based label-free technologies that monitor changes in cell characteristics in response to signal transduction may facilitate fast and accurate real-time readout capabilities for cell-based and other assays. Using label free methods may allow a more direct readout using native physiological or disease relevant settings, without the need to use modified or labelled proteins. These methods may also identify compounds which modulate target activity such as slow acting inhibitors and allosteric modulators, and when used synergistically with traditional biochemical and cell based assays, may aid in identifying a more diverse panel of leads with new mechanisms of action.

As the complexity of drug-able targets increases, it is envisaged that label free technologies will play a complimentary role to existing technologies. Currently, various label-free technologies are available including the xCELLigence (Roche) which is a cellular impedance-based system [17] and the Enspire^®^ (Perkin Elmer) which relies on optical waveguide technology taken from the Corning Epic^®^ system [18]. Further time and evaluation is required to see whether such platforms are indeed suitable for the screening of biologics.

## 7. High Throughput Affinity Screens

Affinity maturation of antibodies and therapeutic proteins is common practice to meet desired criteria and for the potential new drug to bring additional benefit over those already launched. Traditionally, affinity measurements (using surface plasma resonance (SPR) based methods such as BiaCore) [19], are accurately determined for a relatively small panel of antibody candidates (<10). However, the advent of the Octet RED System (ForteBio) [20] with its higher throughput capability has enabled affinity-based screening of much greater numbers of antibodies. This has proven invaluable when biochemical assays have reached the limits of their useful sensitivity and it has become difficult to discriminate between a number of high affinity variants. Although cell based potency assays can be employed to discriminate between the variants, these often require highly purified and refined samples and may take several days to perform. In contrast the Octet RED System can be used to provide information on affinity, kinetics and concentration using relatively crude samples and has proven to be robust and reproducible. Data from the Octet also correlates well with that from BiaCore while the absolute values may have some variation, the rank-ordering of the antibodies remains very consistent.

## 8. Conclusions

The evolution of assay development and screening for bio-pharmaceuticals has been similar to that found in the world of small molecule discovery. Trends and developments in assay technologies have been closely followed and adapted to match scientific and medicinal needs. There has been a clear move away from heterogeneous biochemical and radiometric assays using recombinant proteins to more content and information rich assays with an emphasis on physiological relevance [21]. This has enabled the prosecution of increasingly difficult drug target classes and provides mitigation against the effects of late stage failure rates in the clinic due to a lack of understanding of target engagement in a physiological setting.

Looking forward, high-content, high-throughput imaging systems [22], offer the potential to address some of the challenges faced with defining physiological relevance. These systems are amenable to plate-based screening and allow the measurement of multiple parameters, such as antibody internalization and receptor turnover as well as providing detailed information during phenotypic screening campaigns. Label free technologies also represent a potential methodology to enable physiologically relevant screening, although a more complete understanding or these technologies is required in order to interpret the cellular responses seen with drug addition ,before they can be used successfully in primary screening campaigns.

High throughput screening assays and systems have been designed in order to maximise the sensitivity to antibodies which are often expressed in low nanomolar concentrations. Further adaptations are being made to streamline the drug development process such as the use of high throughput mammalian IgG expression in order to screen in final format. Taken together, these innovations will allow for increased breadth of potential high throughput assays, bringing closer the ultimate goal of running assays on disease relevant cell types in a format which may be more predictive of therapeutic antibody activity in patients.

As described above, from the initial high throughput screens, based on simple ELISA’s through to functional-cell based assays, biologics screening has continued to evolve in order to meet the challenges faced in the identification and development of modern therapeutics. New approaches such as siRNA and antibody drug conjugates will present unique screening challenges in the future. These new modalities will require a highly flexible approach as well as utilization of the most relevant assay platforms for screening to continue to provide meaningful data in the field of biotherapeutics.
